# Tagging Strategies Strongly Affect the Fate of Overexpressed Caveolin-1

**DOI:** 10.1111/tra.12254

**Published:** 2014-12-30

**Authors:** Bing Han, Ajit Tiwari, Anne K Kenworthy

**Affiliations:** 1 Department of Molecular Physiology and Biophysics, Vanderbilt University School of MedicineNashville, TN, USA; 2 Department of Cell and Developmental Biology, Vanderbilt University School of MedicineNashville, TN, USA; 3 Epithelial Biology Program, Vanderbilt University School of MedicineNashville, TN, USA; 4 Chemical and Physical Biology Program, Vanderbilt UniversityNashville, TN, USA

**Keywords:** blue native gel electrophoresis, breast cancer, caveolae, caveolin, detergent-resistant membranes, fluorescent proteins, oligomerization, overexpression, velocity gradient centrifugation

## Abstract

Caveolin-1 (Cav1) is the primary scaffolding protein of caveolae, flask-shaped invaginations of the plasma membrane thought to function in endocytosis, mechanotransduction, signaling and lipid homeostasis. A significant amount of our current knowledge about caveolins and caveolae is derived from studies of transiently overexpressed, C-terminally tagged caveolin proteins. However, how different tags affect the behavior of ectopically expressed Cav1 is still largely unknown. To address this question, we performed a comparative analysis of the subcellular distribution, oligomerization state and detergent resistance of transiently overexpressed Cav1 labeled with three different C-terminal tags (EGFP, mCherry and myc). We show that addition of fluorescent protein tags enhances the aggregation and/or degradation of both wild-type Cav1 and an oligomerization defective P132L mutant. Strikingly, complexes formed by overexpressed Cav1 fusion proteins excluded endogenous Cav1 and Cav2, and the properties of native caveolins were largely preserved even when abnormal aggregates were present in cells. These findings suggest that differences in tagging strategies may be a source of variation in previously published studies of Cav1 and that overexpressed Cav1 may exert functional effects outside of caveolae. They also highlight the need for a critical re-evaluation of current knowledge based on transient overexpression of tagged Cav1.

Caveolae are flask-shaped invaginations of the plasma membrane proposed to function in a series of important processes, such as endocytosis, mechanotransduction, signaling and lipid homeostasis (1). Formation of caveolae requires appropriate expression and organization of the scaffolding protein caveolin-1 (Cav1), a member of the *CAV* gene family that also includes Cav2 and Cav3 (1,2). Cav1 is expressed in multiple cell types and is especially enriched in adipocytes and endothelial cells (3–5). Cav1 has been linked to a number of diseases such as cancer (6–9), pulmonary vascular diseases (3,10,11), lipodystrophy (12), osteoporosis (13), infection (13) and cardiovascular disease (14–16). As a result, the functional properties of Cav1 and caveolae are of interest in multiple areas of research.

Despite extensive investigation, the mechanisms by which Cav1 and caveolae regulate cellular functions remain unclear. For some time, caveolae were believed to be sites of scaffolding of signaling proteins, a process mediated by interactions of proteins containing a putative caveolin-binding motif with Cav1's scaffolding domain (17,18). However, recent structural and bioinformatic analysis indicates that this model is unlikely to be correct (19,20). Recent evidence suggests that Cav1 may instead regulate signaling by an indirect mechanism (21). In addition, Cav1 has been reported to play conflicting roles, including both positively and negatively regulating tumor progression (7,22). Thus, there is currently no consensus model for how Cav1 or caveolae carry out the many functions they have been proposed to regulate.

Transient overexpression has been widely used to study the caveolin proteins and caveolae in the last two decades, and a significant amount of knowledge of Cav1 we now have is based on these studies (23–72). In order to facilitate direct observation of Cav1 dynamics in live cells or study specific caveolin mutants, fluorescent proteins (FPs) (26,27,34,35,43,51,56,58–60,62–65,67–71) or epitope tags (23,26,33,36–38,40,42,47,49,61) are often fused to Cav proteins. Cav1 is a small protein (∼20 kDa), and its proper incorporation into caveolae requires a series of oligomerization events as it traffics from endoplasmic reticulum (ER) to the Golgi complex and finally to the plasma membrane (60). It is thus not surprising that some studies have reported the addition of tags can disrupt the targeting and function of Cav proteins. For example, early reports suggested that the N-terminus of Cav1 is critical for caveolae-mediated uptake processes (67), and N-terminally tagged Cav1 behaves as a dominant negative inhibitor in SV40 infection experiments (68). As a result, subsequent studies fused tags to the C-terminus of Cav proteins (59,60,65,69–71,73). When stably expressed at low levels, C-terminally tagged Cav1 appears to become correctly incorporated into caveolae (60,70,74–76,78). Interestingly, transiently overexpressed Cav1 is sometimes excluded from caveolae (70,73), whereas in other instances overexpression of the protein appears to drive new caveolae formation (78–81). Furthermore, we have observed that the subcellular localization patterns of overexpressed Cav1 also vary depending on how the protein is tagged on its C-terminus and the cellular context (81). These findings suggest that not only overexpression but also the nature of the FP or epitope tags can potentially affect the behavior of transiently expressed exogenous Cav. However, a systematic analysis of how various tags affect the fate of overexpressed Cav proteins is currently lacking.

To address this issue, we conducted a comparative analysis of the subcellular distribution, oligomerization state and detergent resistance of transiently overexpressed Cav1 constructs containing three different tags (EGFP, mCherry and myc). For comparison, we also studied one of the best characterized Cav1 mutant, P132L (80–86). Our results show that overexpressed Cav1-FPs are largely sequestered in irregular complexes that exclude endogenous Cav1 and Cav2 and that the presence of the overexpressed protein has only subtle effects on endogenous caveolins. Furthermore, the nature of the tags differentially affected the behavior of overexpressed exogenous Cav1 and P132L in all of the assays examined. Taken together, our data imply that tagging strategies may represent an underappreciated source of variation in published studies of Cav1 and suggest that overexpressed Cav1 may exert its functional effects outside of caveolae. These findings call for a systematic re-evaluation of results based on transient overexpression of tagged Cav1.

## Results

### The nature of the tag affects the subcellular distribution pattern of overexpressed Cav1

As a model system to study the effects of tagging on transiently overexpressed Cav1, we compared the behavior of wild-type Cav1 and P132L Cav1, a breast cancer associated mutant that is mistrafficked in cells by mechanisms thought to involve defects in the oligomerization of the protein (60,84,86). In contrast to the behavior of wild-type Cav1, P132L forms a mixture of monomers/dimers and high molecular weight oligomers as assessed by velocity centrifugation (84) and blue native-PAGE (BN-PAGE) (87). These reported defects of P132L provide a good benchmark for comparative analysis with wild-type Cav1.

**Figure 1:**
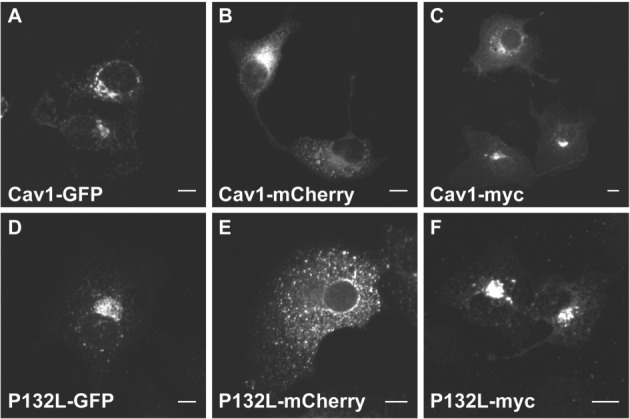
The subcellular distribution of transiently overexpressed wild-type Cav1 and P132L Cav1 fusion proteins differ depending on the tag. COS-7 cells were transiently transfected with (A) Cav1-GFP, (B) Cav1-mCherry, (C) Cav1-myc, (D) P132L-GFP, (E) P132L-mCherry or (F) P132L-myc for 24 h, fixed and imaged. Cells expressing myc-tagged Cav1 constructs were immunostained prior to imaging. Bars, 10 µm.

In a previous study, we found that overexpressed wild-type Cav1 had a similar subcellular distribution as P132L, but that their distributions varied depending on the nature of the tag (EGFP, mCherry or myc) (81). When transiently expressed in COS-7 cells, Cav1-GFP and P132L-GFP primarily accumulated in the perinuclear region (Figure 1A, D) in the majority of cells (81). The localization of Cav1-mCherry and P132L-mCherry was dramatically different from their GFP counterparts (Figure 1B, E). In about 20% of cells, Cav1-mCherry was diffusely distributed on the plasma membrane and in the ER, while 80% of the cells contained a perinuclear pool of Cav1-mCherry and bright fluorescent puncta within the cytoplasm (81). In most of the P132L-mCherry-transfected cells, P132L-mCherry exists as bright fluorescent puncta with in the cytoplasm (81). Two phenotypes were also observed in Cav1-myc-transfected cells. One phenotype showed perinuclear accumulation, and the other discrete puncta (Figure 1C) (81). Here, we generated a P132L-myc construct for comparison. Consistent with a former report, all of the P132L-myc-transfected cells displayed a classical perinuclear accumulation (Figure 1F) (84). In summary, all of the overexpressed tagged wild-type Cav1 or mutant (P132L) constructs display a perinuclear accumulation phenotype to a different degree as summarized in Table 1.

**Table 1:** Summary of the cellular and biochemical features of transiently overexpressed wild-type and P132L mutant Cav1 constructs as a function of different tags. The extent of each feature is represented by the number of stars. The value of an empty star is half the value of a filled star

			Blue -native electrophoresis		Velocity gradient centrifugation (no SDS)			Velocity gradient centrifugation (with SDS)		
	Perinuclear localization	Degradation tendency	HMW band/smear	Smears/bands below endogenous Cav1	8S-like complex	70S-like complex	HMW aggregate	8S-like complex	Monomer/LMW oligomer	DRM affinity
Cav1-GFP	★★★	★☆	★★★	★★	★	None	★★★	★★	★☆	★
Cav1-mCherry	★	☆	★★★	★★	★★	None	★★★	★★☆	☆	★★
Cav1-myc	★☆	None	★★★	★★	★★★	★★	★★	★★★	None	★★★
P132L-GFP	★★★	★	★★	★★★	None	None	★★	None	★★★	☆
P132L-mCherry	None	★★★	None	★★★	None	None	☆	None	★★★	☆
P132L-myc	★★★	None	★★	★★★	None	None	None	None	★★★	None

HMW, high molecular weight; LMW, low molecular weight.

### The oligomerization state of overexpressed Cav1 varies as a function of the tag as reported by BN-PAGE

Because the Cav1 constructs differed in their subcellular distribution, we wondered if they also differ in their oligomerization status. Former studies found that newly synthesized Cav1 is inserted into membranes of the ER and undergoes a series of oligomerization events as it traffics through the secretory pathway (88–90), including the formation of 8S complexes in the ER and 70S complexes in the Golgi complex (60). To test of the ability of overexpressed Cav1 to form oligomers, we used BN-PAGE, a technique that has previously been used to investigate the oligomerization states of proteins including caveolins (87,91,92). This approach can effectively detect oligomerization defects of mutant Cav1 (87). For example, transiently overexpressed P132L Cav1 exists as a mixture of monomers/dimers and high molecular weight oligomers following BN-PAGE (87).

**Figure 2:**
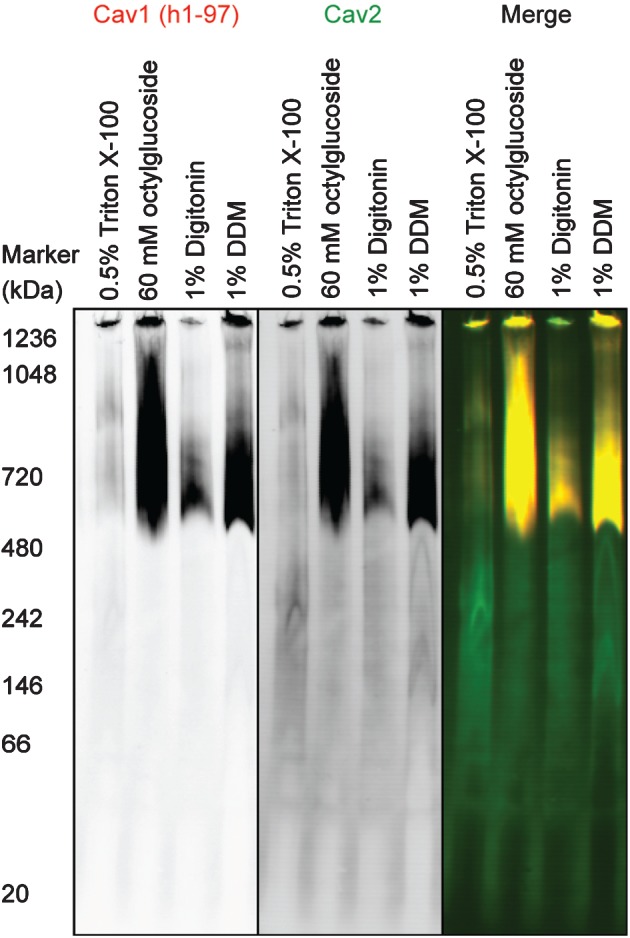
Endogenous Cav1 and Cav2 co-migrate as hetero-oligomers. COS-7 cells were lysed in the indicated detergents and subjected to BN-PAGE followed by western blotting for Cav1 (red) and Cav2 (green).

BN-PAGE separates membrane protein complexes using mild conditions that preserve protein–protein interactions (93). We combined the use of BN-PAGE and dual-color detection of immunoblotted proteins so that we could simultaneously detect two proteins on one membrane. To determine optimal solubilization conditions for these experiments, we compared several different detergents, including 0.5% Triton-X-100, 60 mm octylglucoside, 1% digitonin and 1% n-dodecyl β-D-maltoside (DDM), detergents commonly used for BN-PAGE analyses (87,91,92,94), where 0.5% Triton-X-100 was unable to solubilize Cav1 effectively. However, for all the other conditions tested, endogenous Cav1 was isolated as part of a high molecular weight complex from COS-7 cells (Figure 2). For example, in digitonin-solubilized cells, Cav1 migrates as part of a ∼600 kDa complex. The is very similar in size to previous reports that solubilized Cav1 with octylglucoside (87) or DDM (92) and likely corresponds to the core Cav1 8S unit complexes observed by the velocity gradient fractionation (60). Cav1 is known to form hetero-oligomers with Cav2 (95,96), so we also tested for the presence of Cav2 in these complexes. As expected, endogenous Cav2 perfectly co-migrated with Cav1. Because digitonin solubilization yielded the best resolution of complexes, this condition was chosen for use for further studies.

**Figure 3:**
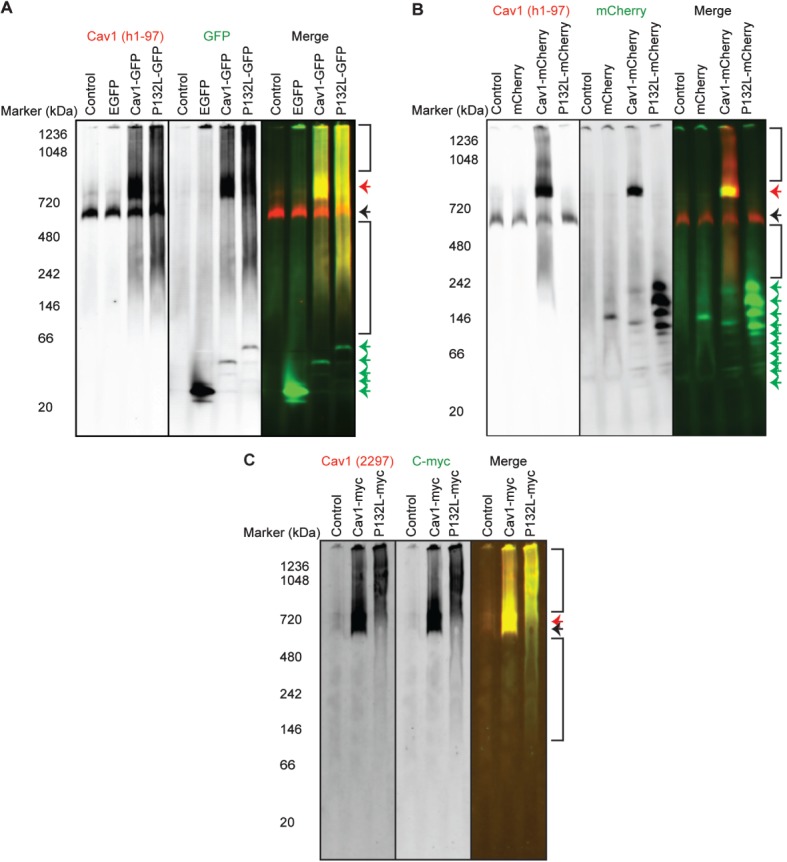
The oligomerization state of overexpressed Cav1 varies as a function of its tag. COS-7 cells expressing the indicated constructs were lysed in digitonin and subjected to BN-PAGE followed by western blotting for Cav1 (red) and either GFP, mCherry or myc (green). A) Cells were either left untransfected (‘control’) or transfected with EGFP, Cav1-GFP or P132L-GFP. B) As in (A) except cells were transfected with the indicated mCherry constructs. C) As in (A) except cells were transfected with Cav1-myc or P132L-myc. Figures are representative of two independent experiments. Red arrows indicate the high molecular weight band positive for both tag antibodies and Cav1 antibodies (h1-97 or 2297). Black arrows indicate the high molecular weight band only positive for Cav1 antibodies (h1-97 or 2297). Green arrows indicate the low molecular weight bands only positive for FP tag antibodies.

We next tested for possible defects in the oligomerization status of overexpressed wild-type Cav1 and P132L with GFP, mCherry or myc tags by using this approach. In untransfected cells (Figure 3A–C, lane 1) or cells expressing either EGFP or mCherry alone (Figure 3A,B, lane 2) as a negative control, endogenous Cav1 ran as a high molecular weight band of ∼600 kDa. In contrast, cells expressing Cav1-GFP and Cav1-mCherry, two discrete high molecular weight bands were observed. The first had a similar mobility to that of endogenous Cav1 and was not recognized by an anti-GFP antibody (Figure 3A,B, lane 3, black arrow). Thus, it likely represents the endogenous Cav1 complex. The second band (Figure 3A,B, lane 3, red arrow) corresponded to a higher molecular weight and was positive for GFP or mCherry, suggesting that it consists of oligomers of Cav1-GFP or Cav1-mCherry. A smear of Cav1- and FPs-positive staining was also seen at lower and higher molecular weights than the band (Figure 3A,B, lane 3, brackets). This suggests that Cav1-GFP and Cav1-mCherry can form discrete high molecular weight oligomers distinct from those containing only endogenous Cav1, as well as oligomers of irregular size. Some FP-positive bands were seen at lower molecular weights that appear to consist of partially degraded forms of Cav1-GFP and Cav1-mCherry (Figure 3A,B, lane 3, green arrows). In contrast, overexpressed Cav1-myc formed a very wide band that overlapped with the position of endogenous Cav1 (Figure 3C, lane 2).

The behavior of the P132L constructs was more variable. For P132L-GFP, we observed smears of Cav1- and GFP-positive staining both above and below the position of the endogenous Cav1 bands (Figure 3A, lane 4, brackets) and GFP-positive/Cav1 negative bands at lower molecular weights (Figure 3A, lane 4, green arrows). Interestingly, the low molecular weight GFP-positive bands were of a different size than observed for Cav1-GFP. Similar to P132L-GFP, P132L-myc also formed smears of Cav1- and myc-positive staining above and below the endogenous Cav1 band (Figure 3C, lane 3, brackets). Finally, for P132L-mCherry, only a series of mCherry-positive bands were detected (Figure 3B, lane 4, green arrows). These bands likely represent aggregates of incompletely degraded P132L-mCherry. This again confirmed the variation between different tags.

The finding that all the wild-type Cav1 constructs form a high molecular band that is distinct from the band containing only endogenous Cav1 strongly suggests that transiently overexpressed Cav1 does not form oligomers with endogenous Cav1, and also does not disrupt the oligomerization of endogenous Cav1. To test this idea further, we carried out additional analysis.

### Oligomers containing overexpressed Cav1 or P132L Cav1 exclude endogenous Cav1 and Cav2

To determine whether endogenous Cav1 is present in the higher molecular weight oligomers or smears enriched in tagged Cav1 or P132L, we took advantage of our previous observation that the C-terminus of Cav1 is not recognized by a C-terminally directed rabbit monoclonal antibody when tagged on the C-terminus (81) (Figure S1). We verified by western blotting that the C-terminal antibody also fails to detect Cav1-GFP even though it recognizes endogenous Cav1 in SDS–PAGE (Figure S1, black arrow). For comparison, a mouse monoclonal antibody that recognizes a region close to the scaffolding domain (2297) detects both endogenous and Cav1-GFP (81). By comparing the relative amounts of labeling of the Cav1-GFP oligomers by the C-terminal antibody and mAb 2297 in western blots of BN-PAGE gels, we could thus determine how much endogenous Cav1 is present in a given band.

**Figure 4:**
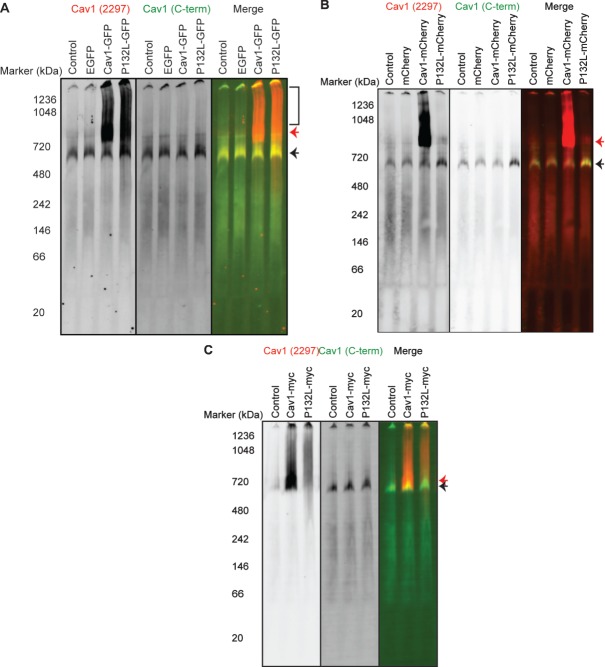
Oligomers containing exogenous tagged Cav1 exclude endogenous Cav1. COS-7 cells expressing the indicated constructs were lysed in digitonin and subjected to BN-PAGE followed by western blotting using antibodies directed against the scaffolding domain (2297, red) or the C-terminus (C-term, green) of Cav1. Cells were either left untransfected (‘control’) or transfected with (A) EGFP, Cav1-GFP or P132L-GFP, (B) mCherry, Cav1-mCherry or P132L-mCherry or (C) Cav1-myc or P132L-myc. Figures are representative of two independent experiments. Red arrows indicate the oligomers of tagged exogenous Cav1. Black arrows indicate the oligomers of endogenous Cav1.

We found that mAb 2297 recognized a band at ∼600 kDa in untransfected cells and cells expressing GFP alone on BN-PAGE gels (Figure 4A), identical to the position of the band detected by the N-terminal Cav1 antibody (Figure 3A). A similar band was also observed in cells expressing Cav1-GFP or Cav1-P132L (Figure 4A, black arrow). mAb 2297 also strongly labeled the ∼800 kDa band observed in cells expressing Cav1-GFP (Figure 4A, red arrow), as well as a high molecular weight smear for both Cav1-GFP and P132L-GFP (Figure 4A, brackets). In contrast, labeling by the C-terminal antibody was confined to the ∼600 kDa band regardless of whether Cav1-GFP or P132L-GFP were overexpressed (Figure 4A, black arrow). Essentially identical results were obtained for cells expressing Cav1-mCherry (Figure 4B) or Cav1-myc (Figure 4C). These data show that detectable levels of endogenous Cav1 are excluded from high molecular weight oligomers containing exogenous tagged Cav1 or P132L.

**Figure 5:**
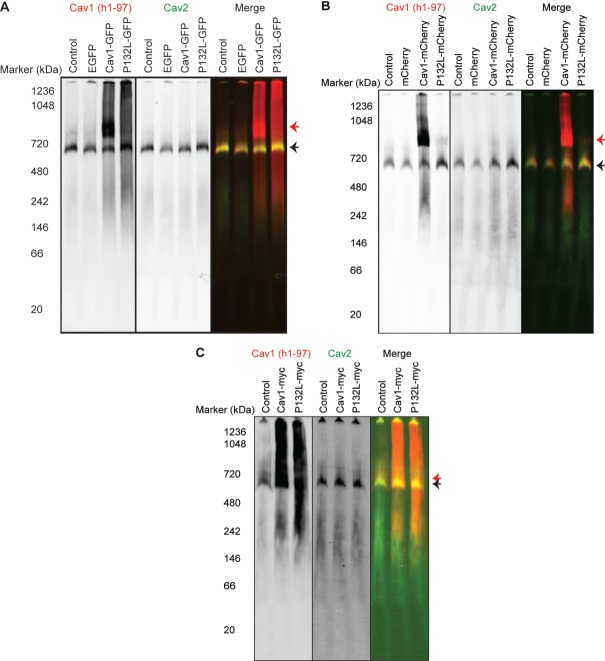
Oligomers containing exogenous tagged Cav1 exclude endogenous Cav2. COS-7 cells expressing the indicated constructs were lysed in digitonin and subjected to BN-PAGE followed by western blotting for Cav1 (h1-97, red) or Cav2 (green). Cells were either left untransfected (‘control’) or transfected with (A) EGFP, Cav1-GFP or P132L-GFP, (B) mCherry, Cav1-mCherry or P132L-mCherry or (C) A Cav1-myc or P132L-myc. Figures are representative of two independent experiments. Red arrows indicate the oligomers of tagged exogenous Cav1. Black arrows indicate the hetero-oligomers of endogenous Cav1 and Cav2.

In addition to forming homo-oligomers, Cav1 forms hetero-oligomers with Cav2 (95,96). To determine whether the Cav1-GFP and P132L-GFP oligomers contained Cav2, we probed western blots of BN-PAGE gels for endogenous Cav2. Levels of Cav2 were similar in all treatments. Cav2 co-migrated with the high molecular weight (∼600 kDa) band of endogenous Cav1 in untransfected cells and cells expressing an empty GFP vector (Figure 5A). Cav2 was found in a similar ∼600 kDa complex with endogenous Cav1 in cells expressing Cav1-GFP and Cav1-P132L (Figure 5A, black arrow). However, Cav2 was excluded from the GFP-positive high molecular weight complex (Figure 5A, red arrow), suggesting that the oligomers formed by Cav1-GFP and Cav1-P132L do not correctly incorporate endogenous Cav2. Similar results were obtained for Cav1-mCherry (Figure 5B) and Cav1-myc (Figure 5C), indicating that this is a generalized defect in complex formation in cells overexpressing tagged Cav1.

### Overexpressed Cav1-FPs form separate complexes from endogenous Cav1/Cav2 hetero-oligomers for at least 4 days

There are several potential explanations for why exogenous and endogenous Cav1 form separate oligomers (Figures 5). In principle, the presence of a tag on Cav1's C-terminus could potentially interfere with its ability to oligomerize with endogenous Cav1, although this appears unlikely given that when expressed at low levels Cav1-GFP is incorporated into caveolae containing endogenous caveolins (60,70,74–76). A second possibility is that there is not enough time for the newly synthesized endogenous Cav1 to oligomerize with exogenous Cav1. Endogenous Cav1 and Cav2 have very long half lives (>36 h) (73) and once Cav1 forms a stable complex, the monomers do not exchange between oligomers freely (64). Similar to many other transient Cav1 expression studies (60,63,64,67,68,74,97), we started our analysis 24 h after transfection, and thus tagged Cav1 may not have the opportunity to interact with the endogenous protein. Finally, the tags themselves could potentially cause the overexpressed protein to aggregate and thus interfere with the normal oligomerization process.

**Figure 6:**
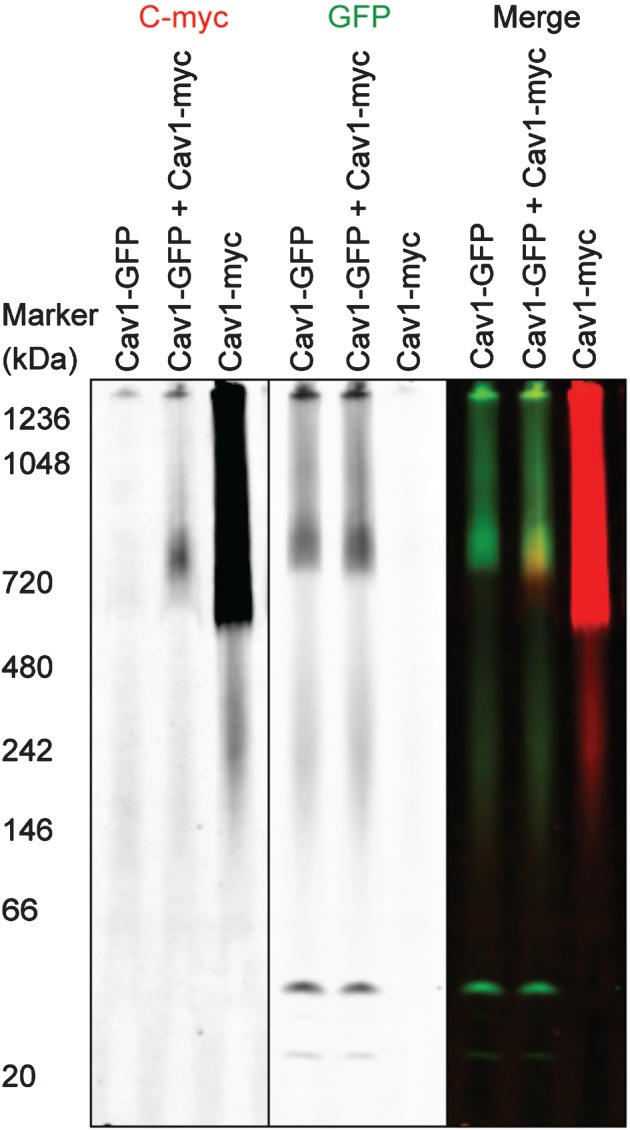
When transiently co-expressed, Cav1 with different tags can form hetero-oligomers. COS-7 cells expressing the indicated constructs were lysed in digitonin and subjected to BN-PAGE followed by western blotting for myc (red) or GFP (green). The signal for the Cav1-myc channel was deliberately overexposed to allow for detection of Cav1-myc signal in the channel corresponding to the co-transfected cells.

To distinguish between these possibilities, we performed two independent experiments. In the first experiment, we transiently co-transfected COS-7 cells with Cav1-GFP and Cav1-myc. As Cav1-GFP is simultaneously expressed with Cav1-myc, we predicted that they should form hybrid oligomers if they are capable of interacting with each other, and that the hybrid oligomer should have a molecular weight intermediate between that of pure Cav1-GFP and Cav1-myc oligomers. Western blot analysis of BN-PAGE gels confirmed this prediction. A hybrid oligomer could be detected with both GFP and myc antibodies in co-transfected cells, and this band was positioned between the pure Cav1-GFP and Cav1-myc oligomer bands (Figure 6). This result demonstrates that the overexpressed Cav1-GFP is capable of oligomerizing with a variant of Cav1 containing only a small epitope tag. However, this experiment does not directly demonstrate whether the Cav1-GFP is capable of oligomerizing with endogenous Cav1 and Cav2.

To test this, we carried out a second experiment to determine whether Cav1-GFP can eventually associate with endogenous Cav1 and Cav2 after longer times of expression. COS-7 cells were not viable following long-term Cav1-GFP overexpression, so, we instead overexpressed Cav1-GFP in HeLa cells. The cells were harvested at different time points for BN-PAGE followed by western blotting analysis up to a total of 4 days post-transfection. As controls, we included a stable HeLa cell line expressing low levels of the same Cav1-GFP construct (76) (Figure S2) and untransfected HeLa cells.

**Figure 7:**
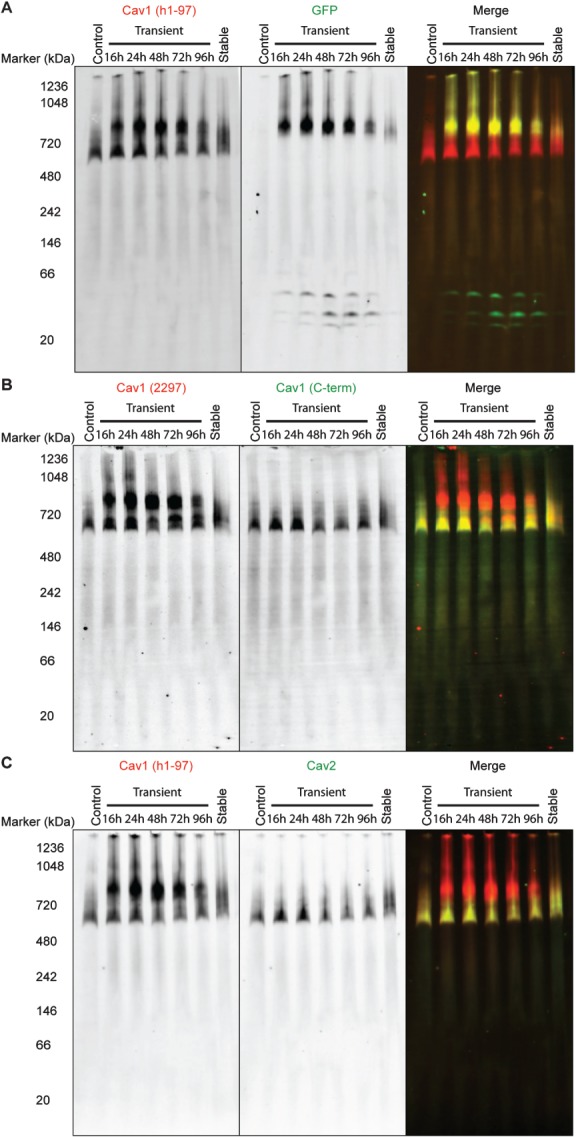
Overexpressed Cav1-GFP fails to oligomerize with endogenous Cav1 and Cav2 for at least 4 days. HeLa cells were either left untransfected (‘control’) or transiently transfected with Cav1-GFP and harvested at the indicated times post-transfection, lysed in digitonin and subjected to BN-PAGE followed by western blotting for (A) Cav1 (h1-97, red) or GFP (green), (B) antibodies directed against the scaffolding domain (2297, red) or the C-terminus (C-term, green) of Cav1, (C) Cav1 (h1-97, red) or Cav2 (green). As a control, a HeLa cell line stably expressing Cav1-GFP was also examined (‘stable’).

As shown in Figure 7A, Cav1-GFP had a different migration pattern in the stable Cav1-GFP HeLa cell line than in its transiently transfected counterpart. For the stably transfected HeLa cell line, the position of the Cav1-GFP-positive band was significantly lower than in transiently transfected cells (Figure 7A). It also co-migrated with endogenous Cav1 and Cav2 (Figure 7B,C). In contrast, we observed two discrete Cav1-positive high molecular weight bands in HeLa cells transiently expressing Cav1-GFP, similar to those observed in COS-7 cells. Only the higher band was recognized by GFP antibody (Figure 7A). The Cav1-GFP-positive band and endogenous Cav1/Cav2 bands remained largely independent over time, although at 72 and 96 h post-transfection, the two bands became somewhat less distinct. These findings suggest that overexpressed Cav1-GFP is sequestered in separate complexes from endogenous Cav1/Cav2 hetero-oligomers for at least 4 days. To further test the possibility that the tags cause the overexpressed protein to aggregate or oligomerize incorrectly, we performed additional studies of the oligomerization state of the protein.

### The oligomerization state of overexpressed Cav1 varies as a function of the tag as detected by velocity gradient centrifugation

**Figure 8:**
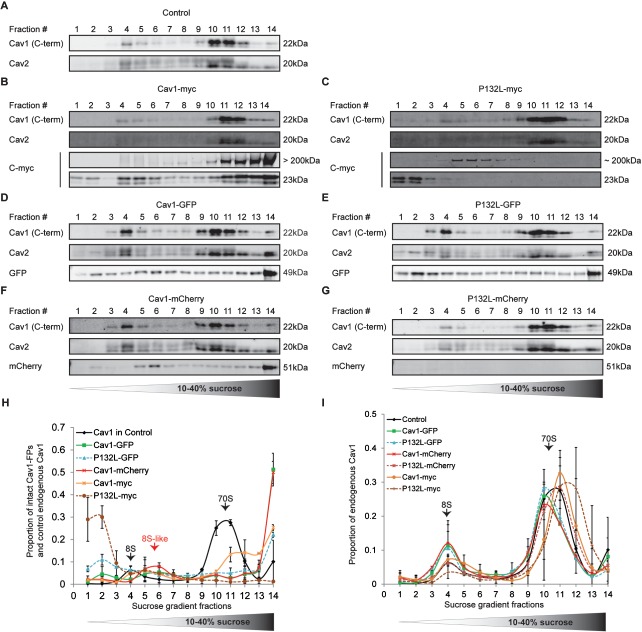
Overexpressed Cav1 and P132L-Cav1 form 8S-like and 70S complexes to differing extents depending on the nature of the tag. A) Untransfected COS-7 cells or cells transiently transfected with (B) Cav1-myc, (C) P132L-myc, (D) Cav1-GFP, (E) P132L-GFP, (F) Cav1-mCherry or (G) P132L-mCherry were lysed in 0.5% Triton-X-100 at room temperature. Extracts were run through 10–40% sucrose velocity gradients and fractions were analyzed by SDS–PAGE/western blot. Densitometry was performed to determine (H) the proportion of the various Cav1 constructs present in each fraction and (I) the proportion of endogenous Cav1 found in each fraction for cells expressing each of the indicated constructs. The positions of the 8S and 70S complexes for endogenous Cav1 are indicated. Bars represent mean ± SD for two independent experiments.

BN-PAGE can effectively detect Cav1/Cav2 hetero-oligomers, but it cannot be used to study very large protein complexes such as 70S Cav1 complexes. To determine if overexpressed Cav1 is incorporated into 8S and 70S complexes correctly, we analyzed cells subjected to 0.5% Triton-X-100 treatment followed by velocity gradient centrifugation (60,84). Control experiments in untransfected cells confirmed that endogenous Cav1 and Cav2 were present in two peaks under these conditions with sizes consistent with the previously described 8S complex (fractions 3–5) and 70S complex (fractions 9–12) (Figure 8A,H,I). Cav1-myc also fractionated into 8S-like and 70S-like complexes, as well as with higher molecular weight complexes (Figure 8B,H). In contrast, Cav1-GFP and Cav1-mCherry were distributed across multiple fractions, and a significant proportion (∼50%) of both Cav1-GFP and Cav1-mCherry was found in high molecular weight complexes in fraction 14 (Figure 8D,F,H). Cav1-GFP and Cav1-mCherry were slightly enriched in fractions 5–7, potentially representing an 8S-like oligomer (Figure 8D,F,H). Cav1-GFP was also found in fractions 2–3 (Figure 8D,F,H) and contained more degraded fragments compared with Cav1-mCherry (Figure S3). No 70S-like complexes were observed in either the Cav1-GFP or Cav1-mCherry fractions. Thus, Cav1-myc, Cav1-GFP and Cav1-mCherry all showed distinct fractionation patterns, and only Cav1-myc associated with both 8S and 70S complexes.

The three P132L constructs also displayed substantial variation as assessed by velocity gradient centrifugation. Consistent with previous reports (84,98), most of the P132L-myc was enriched in fractions 1–2, likely representing a mixture of monomers and dimers (Figure 8C,H). P132L-GFP also failed to incorporate into 8S and 70S complexes, instead forming irregular complexes ranging from monomers to high molecular weight oligomers. A slight enrichment of P132L-GFP was observed in fractions 1–3, suggesting that it maintains some tendency to form low molecular weight oligomers or even monomers. However, a significant proportion of P132L-GFP (∼20%) was also found in high molecular weight complexes in fraction 14 (Figure 8E,H). In most experiments, only degradation products of P132L-mCherry were observed (Figure 8G). In experiments where some intact P132L-mCherry was present, the protein mainly associated with small oligomers (Figure S4A).

We also examined the effects of overexpression of exogenous Cav1 or P132L on the oligomerization state of endogenous caveolins. Similar to the results obtained by BN-PAGE, endogenous Cav1 and Cav2 formed 8S and 70S complexes correctly (Figure 8). However, the proportion of endogenous Cav1 in 8S complexes was slightly increased in cells expressing Cav1-GFP, P132L-GFP and Cav1-mCherry compared with untransfected controls (Figure 8I). Thus, the presence of overexpressed Cav1 or P132L has subtle effects on the organization of endogenous Cav1 and Cav2.

### The high molecular weight aggregates consist of 8S-like complexes for Cav1-FPs and Cav1-myc and small oligomers for P132L-GFP

**Figure 9:**
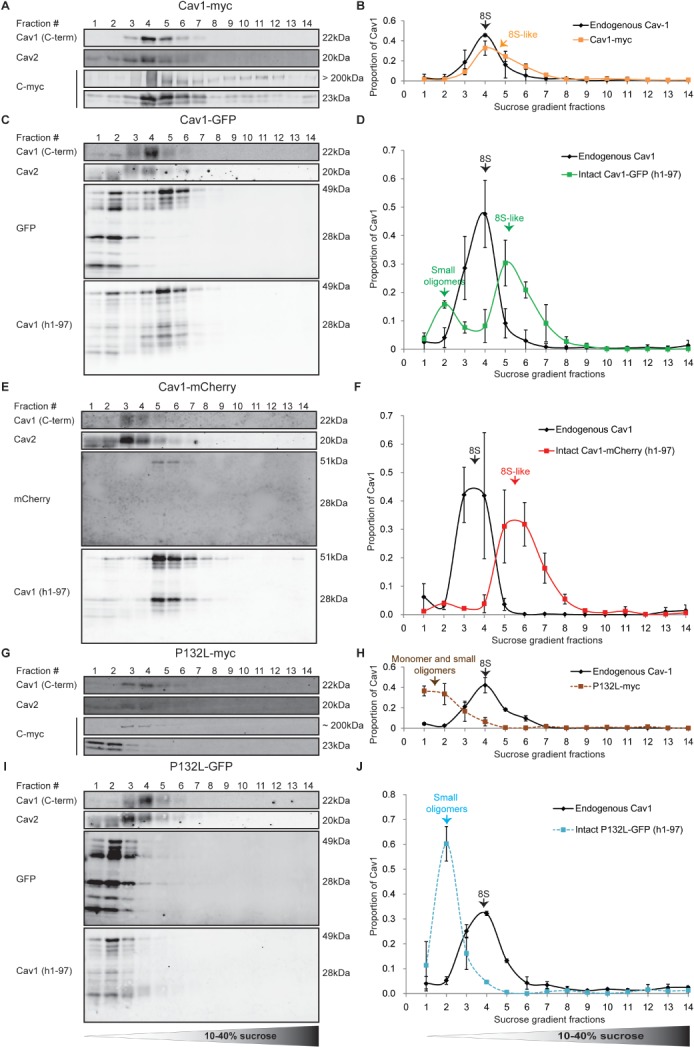
The aggregates of tagged wild-type Cav1 are mainly composed of 8S-like complexes. COS-7 cells expressing (A, B) Cav1-myc, (C, D) Cav1-GFP, (E, F) Cav1-mCherry, (G, H) P132L-myc or (I, J) P132L-GFP were lysed in 0.2% Triton-X-100 and 0.4% SDS at room temperature. Extracts were run through 10–40% sucrose velocity gradients and fractions were analyzed by SDS–PAGE/western blot (A, C, E, G, I) and the levels of overexpressed Cav1 and endogenous Cav1 in each fraction were quantified by densitometry (B, D, F, H, J). The position of the 8S complex containing endogenous Cav1 is indicated by black arrows. Bars represent mean ± SD for two independent experiments.

A large fraction of the Cav1-FPs and Cav1-myc was found in high molecular weight aggregates in the velocity gradient centrifugation experiments (Figure 8). To further investigate the nature of these aggregates, prior to the velocity gradient centrifugation, we lysed the cells with a combined detergent solution containing 0.2% Triton-X-100 and 0.4% SDS previously shown to disassemble the 70S complexes (60). Under these conditions, Cav1-myc and Cav1-mCherry disassembled into 8S-like complexes (Figure 9A, B, E, F), whereas for Cav1-GFP, a combination of monomers or small oligomers and 8S-like complexes was observed (Figure 9C,D). The GFP tag may, thus, partially interfere with the formation of 8S-like oligomers. Consistent with previous findings that P132L tends to oligomerize poorly (60,84), the P132L constructs dissociated into low molecular weight oligomers (Figures 9G–J and S4B). These findings suggest that although both wild-type and P132L form irregular aggregates and high molecular weight oligomers, the aggregates are formed from different building blocks.

### Overexpressed Cav1-FPs do not co-fractionate with DRMs

**Figure 10:**
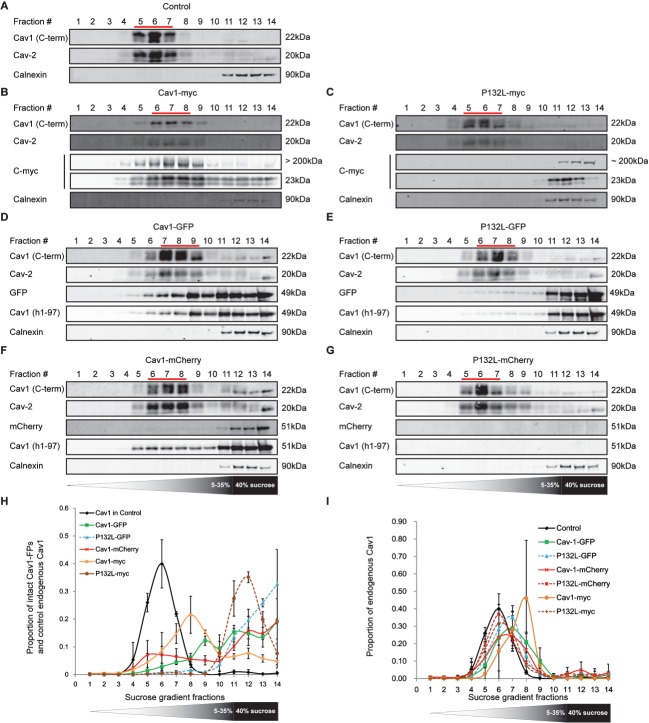
The affinity of overexpressed Cav1 for DRMs differs as a function of the tag. DRMs were isolated from (A) untransfected COS-7 cells or cells transiently transfected with (B) Cav1-myc, (C) P132L-myc, (D) Cav1-GFP, (E) P132L-GFP, (F) Cav1-mCherry or (G) P132L-mCherry. Fractions were analyzed by SDS–PAGE/western blotting. The levels of (H) overexpressed Cav1 and (I) endogenous Cav1 in each fraction were quantified by densitometry. The position of endogenous Cav1 in DRMs is indicated with a red line in (A–F). Bars represent mean ± SD for two independent experiments.

The results of the velocity sucrose gradient centrifugation fractionation analysis indicate that overexpressed Cav1-FPs fail to form 70S complexes properly. To gain further insight into the properties of these various complexes, we asked whether they could associate with detergent-resistant membranes (DRMs), a characteristic feature of Cav1 (60,84,99). We conducted density gradient centrifugation analysis to separate DRM and detergent-soluble membrane. Control experiments in untransfected cells verified that endogenous Cav1 and Cav2 associated with DRMs and that these fractions were distinct from detergent-soluble membranes containing calnexin (Figure 10A). A large fraction of Cav1-myc also associated with DRMs, although the position of this DRM peak was shifted slightly relative to the position of endogenous Cav1 in control cells (Figure 10B,H). In contrast, Cav1-GFP and Cav1-mCherry had only modest affinity for DRMs (Figure 10D,F,H), and P132L constructs were either degraded or essentially completely detergent soluble (Figures 10C,E,G, S4 and S5). These results further emphasize that overexpressed Cav1 and P132L-Cav1 have different fates depending on the type of tag they are fused with.

Some of the effects of transient Cav1 overexpression could potentially arise from alterations in the properties of existing caveolae. To address this possibility, we examined the effect of overexpression of the various Cav1 constructs on the association of endogenous Cav1 and Cav2 with DRMs. Endogenous Cav1 and Cav2 floated in the presence of overexpressed Cav1 (Figure 10I). However, compared with untransfected controls, endogenous Cav1 and Cav2 shifted to higher density fractions in the presence of overexpressed Cav1 (Figure 10I). This suggests that even though tagged Cav1 form complexes distinct from those containing endogenous Cav1 and Cav2, their presence in the cell affects the composition of endogenous caveolae in a manner that alters their buoyant density.

## Discussion

Much of our current knowledge on the properties of Cav1 and caveolae has been derived from studies of tagged, transiently overexpressed Cav1. In order to understand how tagging influences the behavior of the protein, we conducted a systemic comparison of the effects of three C-terminal tags (EGFP, mCherry and myc) on two different Cav1 constructs (wild-type and P132L mutant). We examined several fundamental properties of Cav1, including the ability of tagged Cav1 to oligomerize, associate with DRMs, localize correctly and form complexes with endogenous Cav1 and Cav2.

Our results indicate that the behavior of both wild-type Cav1 and the P132L mutant is strongly affected by the nature of the tag (summarized in Table 1). When labeled with an epitope tag (myc), Cav1 maintained the most natural phenotype. In contrast, addition of FP tags enhanced the aggregation and/or degradation of both wild-type and P132L mutant Cav1 constructs. In general, the addition of an EGFP tag at the C-terminus tended to cause both wild-type Cav1 and the P132L mutant to aggregate more strongly than did mCherry (Table 1). For the case of wild-type Cav1, this does not appear to be the result of defects in 8S complex formation, as the irregular aggregates could be resolved at least partially into 8S complexes (Figure 9). Instead, it may reflect a tendency of FPs to drive the formation of higher-order aggregates when attached to proteins that themselves form homo-oligomers (100). Recently, FP-driven clustering of oligomeric proteins has been reported to occur even when the FPs appear to themselves exist primarily as monomers (100). This effect may be diluted when low levels of tagged Cav1 are stably expressed in cells expressing endogenous Cav1 due to mixing of the tagged and endogenous proteins within the same complexes. Consistent with this, control experiments verified that Cav1-GFP formed mixed complexes with endogenous Cav1 in a widely studied HeLa cell line stably expressing low levels of Cav1-GFP (Figure 7).

An important implication of our findings is that some of the reported phenotypes of overexpressed Cav1 could potentially be driven by the tags, rather than representing the true behavior of the protein. As one example, in our experiments, FP-tagged Cav1 and P132L were partially degraded into fragments that were positive for either GFP or Cav1. Such fragments may be detected directly or indirectly by microscopy and may contribute to misleading results. The behavior of mCherry-tagged P132L provides a good example of such behavior: very little intact protein was detected in most biochemical experiments, yet mCherry-positive puncta were readily detected in cells (Figure 1, Table 1). Thus, multiple tags should be tested when Cav1 overexpression systems are used in the future in order to distinguish true phenotypes from unspecific effects introduced by the tags.

Our results also clearly show that overexpressed FP-tagged Cav1 or P132L form complexes that exclude detectable amounts of endogenous Cav1 and Cav2. For example, our BN-PAGE results indicated that complexes containing endogenous Cav1 and Cav2 are distinct from tagged Cav1 oligomers (Figures 5 and 7). The ∼600 kDa complexes containing endogenous Cav1 and Cav2 observed in BN-PAGE likely correspond to 8S Cav1/Cav2 hetero-oligomers. Thus, the majority of exogenous and endogenous Cav1 form separate complexes at the earliest stages of complex formation (although we cannot exclude the possibility that low levels of endogenous Cav1 and Cav2 interact with the exogenous proteins). Our velocity gradient and DRMs fractionation experiments further confirmed the independence of exogenous Cav1 and endogenous Cav1/Cav2: they displayed totally different distribution patterns. Furthermore, they were maintained in separate complexes for at least 4 days after transfection. These findings are consistent with previous reports that transiently overexpressed Cav1 does not always become fully incorporated into caveolae (70,73). However, it is important to note that in some cases, overexpression of Cav1 results in the formation of additional caveolae, even without co-expression of other caveolae-stabilizing proteins such as the cavins (78–80). Clearly, more work is needed to determine under what conditions Cav1 is limiting for caveolae formation and how caveolae assembly is affected by specific Cav1 tagging procedures.

The relationship between endogenous and exogenous Cav1 has been investigated in several previous studies (35,62,67). Here, we also examined the effect of Cav1 overexpression on the properties of endogenous Cav1 and Cav2. Remarkably, only subtle changes in the behaviors of the endogenous proteins were observed. For example, in the presence of overexpressed Cav1, the buoyant density of endogenous Cav1 and Cav2 shifted slightly as assessed by DRM analysis (Figure 10). Interestingly, a similar phenomenon was also reported in a study of caveolin mutants, and was proposed to represent a phenotype induced by the mutation (67). Our current results suggest that this shift may represent a general phenomenon rather than a specific mutant phenotype.

In summary, our results indicate that tagging of Cav1 or its mutants can lead to dramatically different properties of the protein in the context of transient overexpression systems. Tagging with FPs in particular appears to drive the protein to form irregular aggregates that are either rapidly degraded or that fail to incorporate into caveolae correctly. These aggregates exclude endogenous Cav1 and Cav2. Furthermore, the biochemical properties of endogenous caveolins are largely preserved when these abnormal aggregates are present. This suggests that exogenous Cav1 may exert effects outside of caveolae that drive many of the phenotypes previously associated with Cav1 overexpression. Differences in Cav1 tagging could also represent a previously unappreciated source of variation in published studies. Given these findings, it will be important to re-evaluate current knowledge based on the transient overexpression of tagged Cav1, especially in the case where the fate of the overexpressed protein was not specifically documented.

## Materials and Methods

### Cells, constructs and antibodies

COS-7 and HeLa cells (obtained from ATCC) were cultured in DMEM containing 10% fetal bovine serum, 1% Pen/Strep at 37°C and 5% CO_2_. Cav1-GFP stably transfected HeLa cells (74) were kindly provided by Dr. Benjamin J. Nichols (Medical Research Council Laboratory of Molecular Biology), and were maintained in DMEM containing 10% fetal bovine serum, 1% Pen/Strep supplemented with 0.4 mg/mL G418 (Sigma) at 37°C and 5% CO_2_. Cells were plated 2 days prior to experiments, and transient transfections were performed using FuGENE 6 as per the manufacturer's instructions (Roche Diagnostics). One microgram of DNA was used for individual wells of six-well plates and 6 µg was used for 10 cm dishes. Unless otherwise indicated, cells were transfected 1 day prior to experiments. Cav1-GFP, P132L-GFP, Cav1-mCherry, P132L-mCherry and Cav1-myc were constructed as described previously (81). P132L Cav1-Myc was generated by site-directed mutagenesis of Cav1-Myc, using following set of primers (eurofins mwg/operon): forward primer: 5′-CATCTGGGCAGTTGTACTATGCATTA-3′ and reverse primer 5′-TAATGCATAGTACAACTGCCCAGATG-3′ using standard techniques. Clones positive for P132L Cav1-myc were validated by DNA sequencing. Rabbit anti-Cav1 polyclonal antibody (referred to here as pAb h1-97; catalog number 610059), mouse monoclonal (mAb) anti-Cav1 clone 2297 (mAb 2297, catalog number 610406), mouse anti-Cav2 mAb (mAb Cav2, catalog number 610684) and mouse anti-calnexin mAb (catalog number 610523) were obtained from BD Transduction Laboratories. Rabbit anti-C-terminal Cav1 mAb (C-term, catalog number 1249-1) was obtained from Epitomics. Mouse anti-GFP mAb (catalog number 632381) was obtained from Clontech. Mouse anti-mCherry mAb (catalog number NBP1-96752) was obtained from NOVUS. Rabbit anti c-Myc pAb (catalog number sc-789) was obtained from Santa Cruz Biotechnology (for western blotting). For immunofluorescence assays, mouse anti c-Myc mAb (9B11) (catalog number 2276) was obtained from Cell Signaling Technology. Fluorescently conjugated secondary antibodies and blocking buffer were obtained from LI-COR Biosciences (for Western blotting). For immunofluorescence assays, Alexa-labeled secondary antibodies were obtained from Life Technologies.

### Immunoflorescence microscopy

COS-7 cells grown on glass coverslips were transfected with Cav1 constructs and processed for immunofluorescence 24–30 h after transfection. The transfected cells were rinsed twice and fixed for 15 min in 4% Paraformaldehyde (PFA) in PBS. After rinsing in PBS, the cells were permeabilized and blocked for 1 h at room temperature in blocking buffer composed of 0.1% Triton-X-100 in PBS containing 5% glycine and 5% normal goat or donkey serum. The cells were either left unstained (for GFP/mCherry tagged constructs) or stained with anti-Myc (1:100 dilution in blocking buffer) for 2 h at room temperature. After rinsing in PBS, coverslips were incubated for 1 h in a 1:200 dilution of either Alexa 488- or Alexa 546-conjugated secondary antibodies, rinsed and mounted using Prolong Gold antifade reagent (Life Technologies). Confocal Z-stacks were acquired using a Zeiss LSM 510 confocal microscope with a 40×1.4 NA Zeiss Plan-Neofluar oil immersion objective. For presentation purposes, Z-stacks were combined using the Z projection tool of ImageJ and image contrast was adjusted using Photoshop.

### Electrophoresis and western blotting

BN-PAGE was conducted using the NativePAGE™ Bis-Tris Gel System (Life Technologies). Cell lysis buffer (NativePAGE 1× sample buffer, complete protease inhibitor cocktail from Roche and either 1% digitonin, 0.5% Triton-X-100-100, 60 mm octylglucoside or 1% DDM) was made according to the NativePAGE Sample Prep Kit's handbook. Cells were lysed at 4°C for 30 min. Then, a 30-min centrifugation at 16 100 × g (centrifuge 5415D, Eppendorf) at 4° C was conducted. The pellet was discarded and the supernatant was used for the following analysis. Protein concentrations were determined by using a BCA Assay Kit from Thermo Scientific. 4–16% NativePAGE gels (Life Technologies) were used for the protein separation. Equal amounts of protein were loaded on the same gel as determined by BCA (typically between 15 and 18 µg for each lane). NativeMark™ unstained protein standards (Life Technologies) were used to evaluate the molecular weight.

SDS–PAGE was conducted by using Novex® NuPAGE® SDS–PAGE Gel System (Life Technologies). NuPAGE 4–12% Bis-Tris gels (Life Technologies) were used for the protein separation. SeeBlue® Pre-stained Protein Standard (Life Technologies) was used to evaluate the molecular weight.

A Mini Trans-Blot® Electrophoretic Transfer Cell (Bio-Rad) was used for the electrophoretic transfer. PVDF membranes (from Millipore) were de-stained with methanol (for BN-PAGE). Blots were probed with the indicated primary antibodies followed by fluorescent secondaries and the fluorescence signal was detected using a LI-COR Odyssey infrared imaging system (LI-COR Biosciences.) Quantification of western blot images was performed using ImageJ.

### Velocity gradient centrifugation

Velocity gradient centrifugation was adapted from a previously described method (60). About 2 × 10^6^ COS-7 cells were lysed at room temperature for 20 min in 330 μL of 0.5% Triton-X-100 (or 0.4% SDS and 0.2% Triton-X-100) in TNE [100 mm NaCl, 20 mm Tris–HCl pH 7.5 and 5 mm ethylenediaminetetraacetic acid (EDTA)] buffer, supplemented with ‘Complete’ protease inhibitors cocktail (Roche). Post-nuclear supernatants (PNSs) were prepared by a 5-min centrifugation at 1100 × ***g***. Three hundred microliters of the PNS was loaded onto linear 10–40% sucrose gradients containing 0.5% Triton-X-100, 20 mm Tris–HCl pH 7.5, 100 mm NaCl, 5 mm EDTA and protease inhibitors cocktail. After centrifugation in an SW55 rotor (Optima™ LE-80K Ultracentrifuge, Beckman) for 5 h at 48 000 rpm (279 232.1 × g) and 4° C, fourteen 360 μL fractions were collected from the top and analyzed by SDS–PAGE/western blot with an equal loading volume. Western blots were imaged and quantified as indicated above.

### Preparation of Caveolae-enriched membrane fractions

Preparation of caveolae-enriched membrane fractions was adapted from previously described protocols (60,84,101,102). Specifically, about 4 × 10^6^ COS-7 cells were suspended in 300 μL of cold 0.5% Triton-X-100 in TNE [100 mm NaCl, 20 mm Tris–HCl pH 7.5 and 5 mm EDTA], supplemented with ‘Complete’ protease inhibitors cocktail (Roche). Homogenization was performed in a cold room using pre-cooled equipment by passing the cell solution 10 times through a 1-mL syringe with a 27 gauge stainless steel needle (BD Biosciences). The homogenate was adjusted to about 40% sucrose by the addition of 700 μL of 60% sucrose prepared in TNE and placed at the bottom of an ultracentrifuge tube. A 5–30% linear sucrose gradient was formed above the homogenate and centrifuged at 40 100 rpm 194 882 × g and 4° C for 16 h in a SW55 rotor (Optima LE-80K Ultracentrifuge, Beckman). Fourteen 360 μL fractions were collected from the top and analyzed by SDS–PAGE/western blot with an equal loading volume. Western blots were images and quantified as indicated above.

## Supporting Information

**Figure S1: The C-terminus of endogenous Cav1, but not Cav1-GFP, is recognized by a C-terminal Cav1 antibody by western blotting**. COS-7 cells were left untransfected (‘control’) or transfected with the indicated constructs. The day after transfection, cells were lysed and SDS–PAGE and western blotting were performed using an N-terminally directed Cav1 antibody (h1-97), a GFP antibody or a C-terminally directed Cav1 antibody. The position of endogenous Cav1 is indicated by the arrow. This information allows us to use a C-terminally directed antibody to test for the presence of endogenous caveolin in complexes containing Cav1-FPs. This figure is associated with Figure.

**Figure S2: Comparison of amounts of Cav1-GFP expression in transiently transfected HeLa cells versus a HeLa cell line stably expressing low levels of Cav1-GFP**. HeLa cells transiently transfected with Cav1-GFP (‘T’) or stably expressing low levels of Cav1-GFP (‘S’) were lysed and SDS–PAGE was performed followed by western blotting with an N-terminally directed Cav1 antibody (Cav1 h1-97) or GFP antibody. The positions of Cav1-GFP and endogenous Cav1 are indicated by arrows. Similar levels of Cav2 were detected in both sets of cells. β-tubulin was used as a loading control. This result compares the expression level of Cav1-GFP in stably transfected HeLa cell and transiently transfected HeLa cells relative to endogenous Cav1. This figure is associated with Figure

**Figure S3: Overexpressed Cav1 and P132L-Cav1 form 8S-like and 70S complexes to differing extents depending on the nature of the tag**. COS-7 cells transiently transfected with (A) Cav1-GFP, (B) P132L-GFP, (C) Cav1-mCherry and (D) P132L-mCherry were lysed in 0.5% Triton-X-100 at room temperature. Extracts were run through 10–40% sucrose velocity gradients and fractions were analyzed by SDS–PAGE/western blot. This figure shows full blots for Figure, which include the degradation products for FP-tagged Cav1 and P132L.

**Figure S4: Biochemical analysis of intact P132L-mCherry**. A) In cells where intact P132L-mCherry is present, the protein forms small oligomers as assessed by velocity gradient centrifugation of cells lysed in 0.5% Triton-X-100. B) In cells lysed with a combination of 0.4% SDS and 0.2% Triton-X-100, intact P132L-mCherry fractionates as small oligomers. (C) Unlike endogenous Cav1, intact P132L-mCherry is primarily found in detergent-soluble fractions. In most experiments, P132L-mCherry was only present as a degradation product, suggesting that it is rapidly degraded. However, in a subset of experiments, some intact P132L-mCherry could be detected. Several of the biochemical properties of intact P132L-mCherry are described here. This figure is associated with associated with Figures, S3 and S5.

**Figure S5: The affinity of overexpressed Cav1 for DRMs differs as a function of the tag**. DRMs were isolated from COS-7 cells transiently expressing (A) Cav1-GFP, (B) P132L-GFP, (C) Cav1-mCherry and (D) P132L-mCherry, and fractions were analyzed by SDS–PAGE/western blotting. The position of endogenous Cav1 in DRMs is indicated for each blot with a red line. This figure shows full blots for Figure, which include the degradation products for FP-tagged Cav1 and P132L.
