# Supplementary material for: Tagging Strategies Strongly Affect the Fate of Overexpressed Caveolin-1
Source: Traffic. 2014 Dec 30;16(4):417–38. doi: 10.1111/tra.12254 (PMC4440517; doi:10.1111/tra.12254)
Supplement: Supplementary file 1 — Figure S1: The C-terminus of endogenous Cav1, but not Cav1-GFP, is recognized by a C-terminal Cav1 antibody by western blotting. COS-7 cells were left untransfected (‘control’) or transfected with the indicated constructs. The day after transfection, cells were lysed and SDS–PAGE and western blotting were performed using an N-terminally directed Cav1 antibody (h1-97), a GFP antibody or a C-terminally directed Cav1 antibody. The position of endogenous Cav1 is indicated by the arrow. This information allows us to use a C-terminally directed antibody to test for the presence of endogenous caveolin in complexes containing Cav1-FPs. This figure is associated with Figure. [file tra0016-0417-sd1.docx]

**
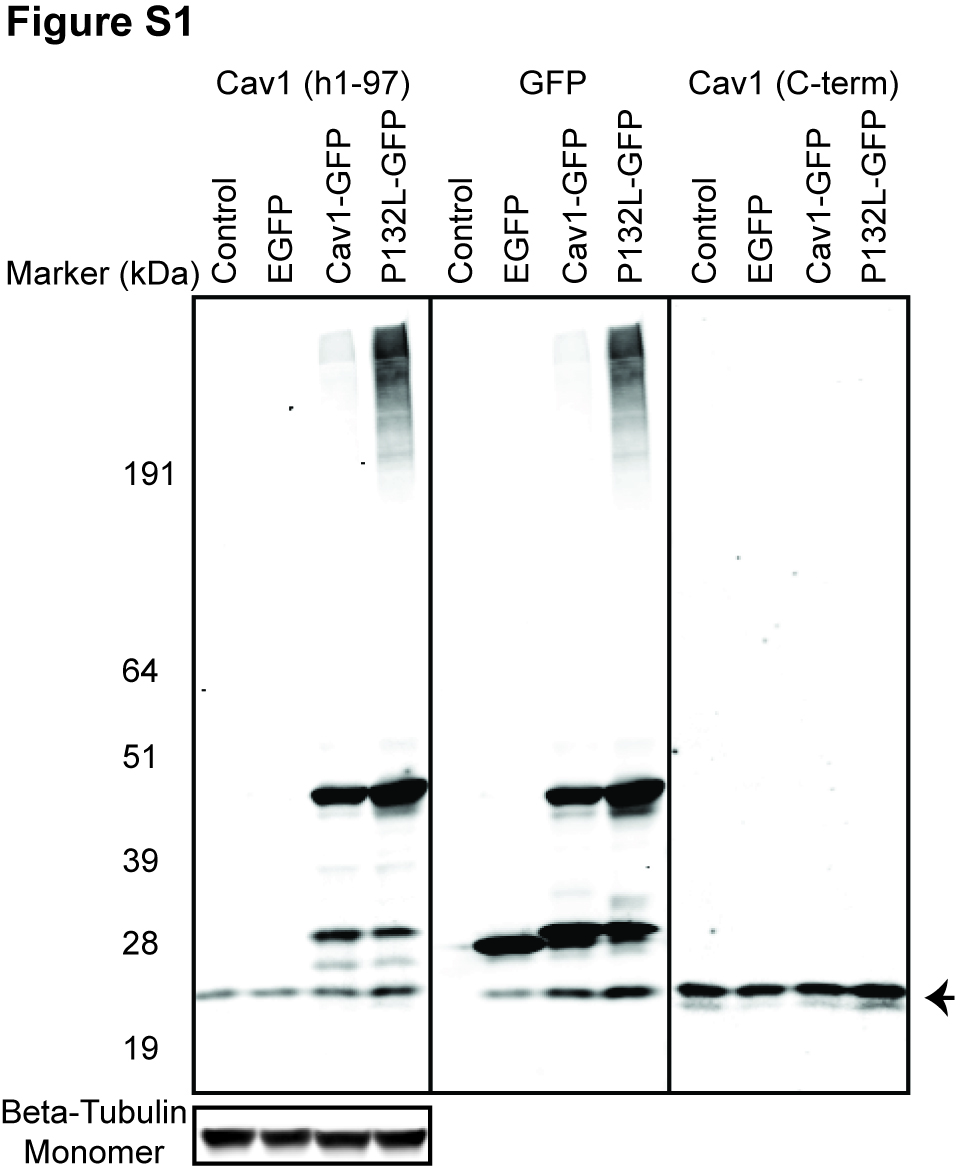
**

**Supplementary Figure 1 (associated with Figure 4). The C-terminus of endogenous Cav1, but not Cav1-GFP is recognized by a C-terminal caveolin-1 antibody by Western blotting.** COS-7 were left untransfected (“control”) or transfected with the indicated constructs. The day after transfection, cells were lysed, and SDS-PAGE and Western blotting was performed using an N-terminally directed Cav1 antibody (h1-97), a GFP antibody, or a C-terminally directed Cav1 antibody. The position of endogenous Cav1 is indicated by the arrow.

This information allows us to use a C-terminally directed antibody to test for the presence of endogenous caveolin in complexes containing Cav1-FPs.
