# Supplementary material for: Tagging Strategies Strongly Affect the Fate of Overexpressed Caveolin-1
Source: Traffic. 2014 Dec 30;16(4):417–38. doi: 10.1111/tra.12254 (PMC4440517; doi:10.1111/tra.12254)
Supplement: Supplementary file 2 — Figure S2: Comparison of amounts of Cav1-GFP expression in transiently transfected HeLa cells versus a HeLa cell line stably expressing low levels of Cav1-GFP. HeLa cells transiently transfected with Cav1-GFP (‘T’) or stably expressing low levels of Cav1-GFP (‘S’) were lysed and SDS–PAGE was performed followed by western blotting with an N-terminally directed Cav1 antibody (Cav1 h1-97) or GFP antibody. The positions of Cav1-GFP and endogenous Cav1 are indicated by arrows. Similar levels of Cav2 were detected in both sets of cells. β-tubulin was used as a loading control. This result compares the expression level of Cav1-GFP in stably transfected HeLa cell and transiently transfected HeLa cells relative to endogenous Cav1. This figure is associated with Figure [file tra0016-0417-sd2.docx]

**
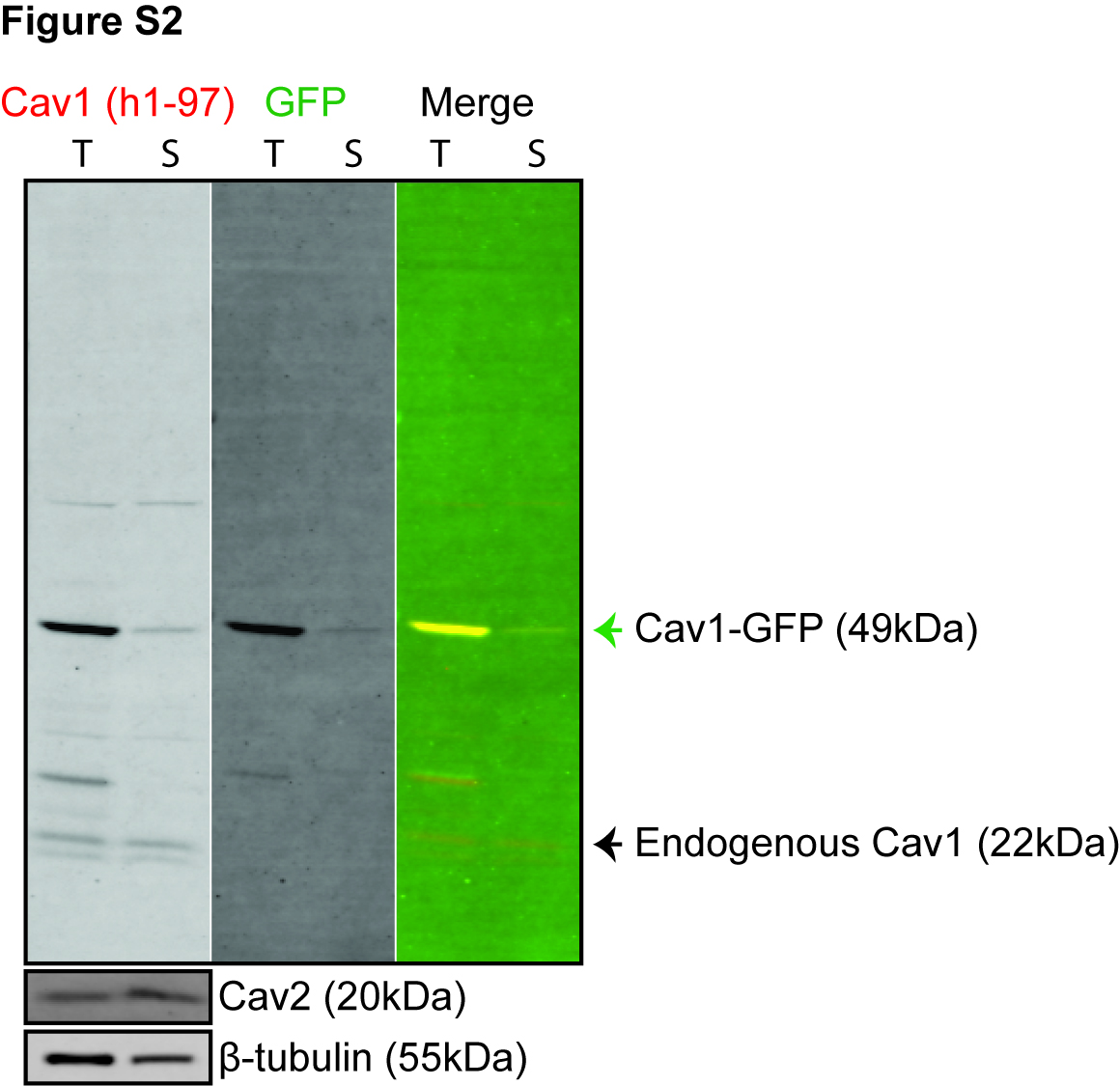
**

**Supplementary Figure 2 (associated with Figure 7). Comparison of amounts of Cav1-GFP expression in transiently transfected HeLa cells versus a HeLa cell line stably expressing low levels of Cav1-GFP.** HeLa cells transiently transfected with Cav1-GFP (“T”) or stably expressing low levels of Cav1-GFP (“S”) were lysed and SDS-PAGE was performed followed by Western blotting with an N-terminally directed Cav1 antibody (Cav1 h1-97) or GFP antibody. The positions of Cav1-GFP and endogenous Cav1 are indicated by arrows. Similar levels of Cav2 were detected in both sets of cells. β-tubulin was used as a loading control.

This result compares the expression level of Cav1-GFP in stably transfected HeLa cell and transiently transfected HeLa cells relative to endogenous Cav1.
