# Supplementary material for: Tagging Strategies Strongly Affect the Fate of Overexpressed Caveolin-1
Source: Traffic. 2014 Dec 30;16(4):417–38. doi: 10.1111/tra.12254 (PMC4440517; doi:10.1111/tra.12254)
Supplement: Supplementary file 3 — Figure S3: Overexpressed Cav1 and P132L-Cav1 form 8S-like and 70S complexes to differing extents depending on the nature of the tag. COS-7 cells transiently transfected with (A) Cav1-GFP, (B) P132L-GFP, (C) Cav1-mCherry and (D) P132L-mCherry were lysed in 0.5% Triton-X-100 at room temperature. Extracts were run through 10–40% sucrose velocity gradients and fractions were analyzed by SDS–PAGE/western blot. This figure shows full blots for Figure, which include the degradation products for FP-tagged Cav1 and P132L. [file tra0016-0417-sd3.docx]

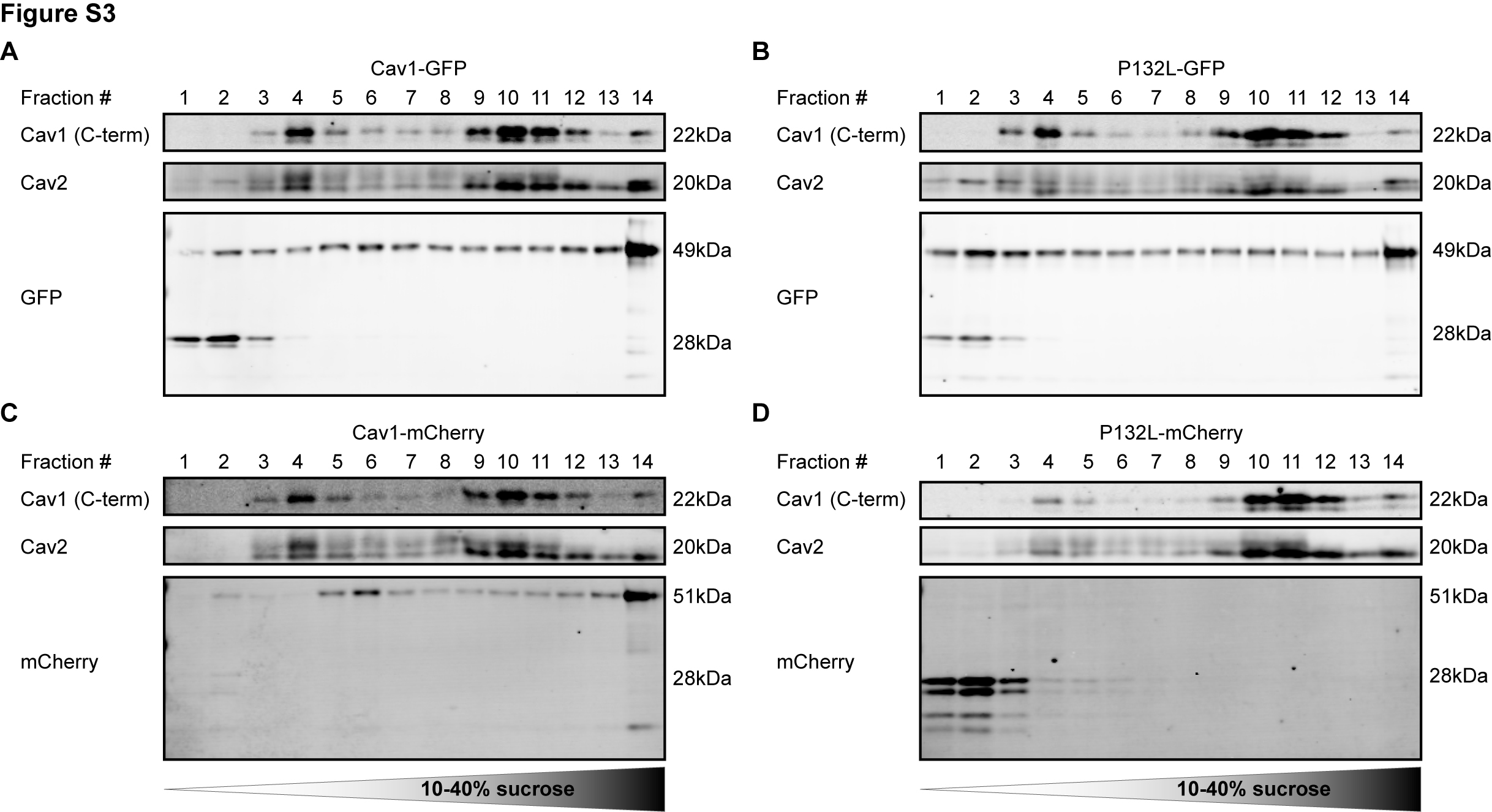


**Supplementary Figure 3 (associated with Figure 8). Overexpressed Cav1 and P132L-Cav1 form 8S-like and 70S complexes to differing extents depending on the nature of the tag.** COS-7 cells transiently transfected with **(A)** Cav1-GFP, **(B)** P132L-GFP, **(C)** Cav1-mCherry, **(D)** P132L-mCherry were lysed in 0.5% Triton X-100 at room temperature. Extracts were run through 10-40% sucrose velocity gradients and fractions were analyzed by SDS-PAGE/Western blot.

This figure shows full blots for Figure 8, which include the degradation products for FP tagged Cav1 and P132L.
