# Supplementary material for: Tagging Strategies Strongly Affect the Fate of Overexpressed Caveolin-1
Source: Traffic. 2014 Dec 30;16(4):417–38. doi: 10.1111/tra.12254 (PMC4440517; doi:10.1111/tra.12254)
Supplement: Supplementary file 4 — Figure S4: Biochemical analysis of intact P132L-mCherry. A) In cells where intact P132L-mCherry is present, the protein forms small oligomers as assessed by velocity gradient centrifugation of cells lysed in 0.5% Triton-X-100. B) In cells lysed with a combination of 0.4% SDS and 0.2% Triton-X-100, intact P132L-mCherry fractionates as small oligomers. (C) Unlike endogenous Cav1, intact P132L-mCherry is primarily found in detergent-soluble fractions. In most experiments, P132L-mCherry was only present as a degradation product, suggesting that it is rapidly degraded. However, in a subset of experiments, some intact P132L-mCherry could be detected. Several of the biochemical properties of intact P132L-mCherry are described here. This figure is associated with associated with Figures, S3 and S5. [file tra0016-0417-sd4.docx]

**
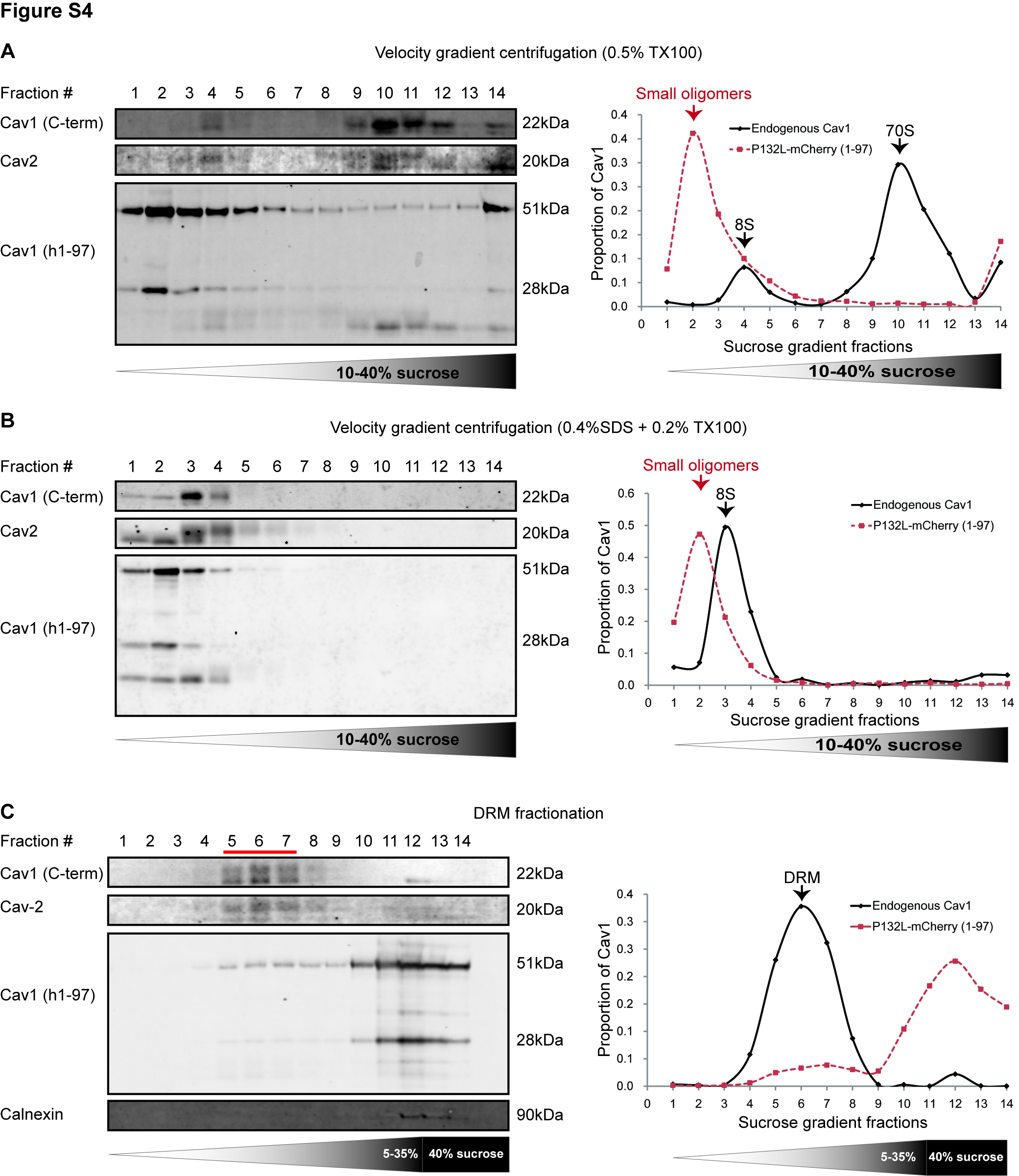
**

**Supplementary Figure 4 (associated with Figures 8, 9, 10 and Figure S3, 5). Biochemical analysis of intact P132L-mCherry. (A)** In cells where intact P132L-mCherry is present, the protein forms small oligomers as assessed by velocity gradient centrifugation of cells lysed in 0.5% Triton X-100. **(B)** In cells lysed with a combination of 0.4% SDS and 0.2% TX-100, intact P132L-mCherry fractionates as small oligomers. **(C)** Unlike endogenous caveolin-1, intact P132L-mCherry is primarily found in detergent-soluble fractions.

In most experiments, P132L-mCherry was only present as a degradation product, suggesting it is rapidly degraded. However, in a subset of experiments some intact P132L-mCherry could be detected. Several of the biochemical properties of intact P132L-mCherry are described here.
