# Supplementary material for: Tagging Strategies Strongly Affect the Fate of Overexpressed Caveolin-1
Source: Traffic. 2014 Dec 30;16(4):417–38. doi: 10.1111/tra.12254 (PMC4440517; doi:10.1111/tra.12254)
Supplement: Supplementary file 5 — Figure S5: The affinity of overexpressed Cav1 for DRMs differs as a function of the tag. DRMs were isolated from COS-7 cells transiently expressing (A) Cav1-GFP, (B) P132L-GFP, (C) Cav1-mCherry and (D) P132L-mCherry, and fractions were analyzed by SDS–PAGE/western blotting. The position of endogenous Cav1 in DRMs is indicated for each blot with a red line. This figure shows full blots for Figure, which include the degradation products for FP-tagged Cav1 and P132L. [file tra0016-0417-sd5.docx]

**
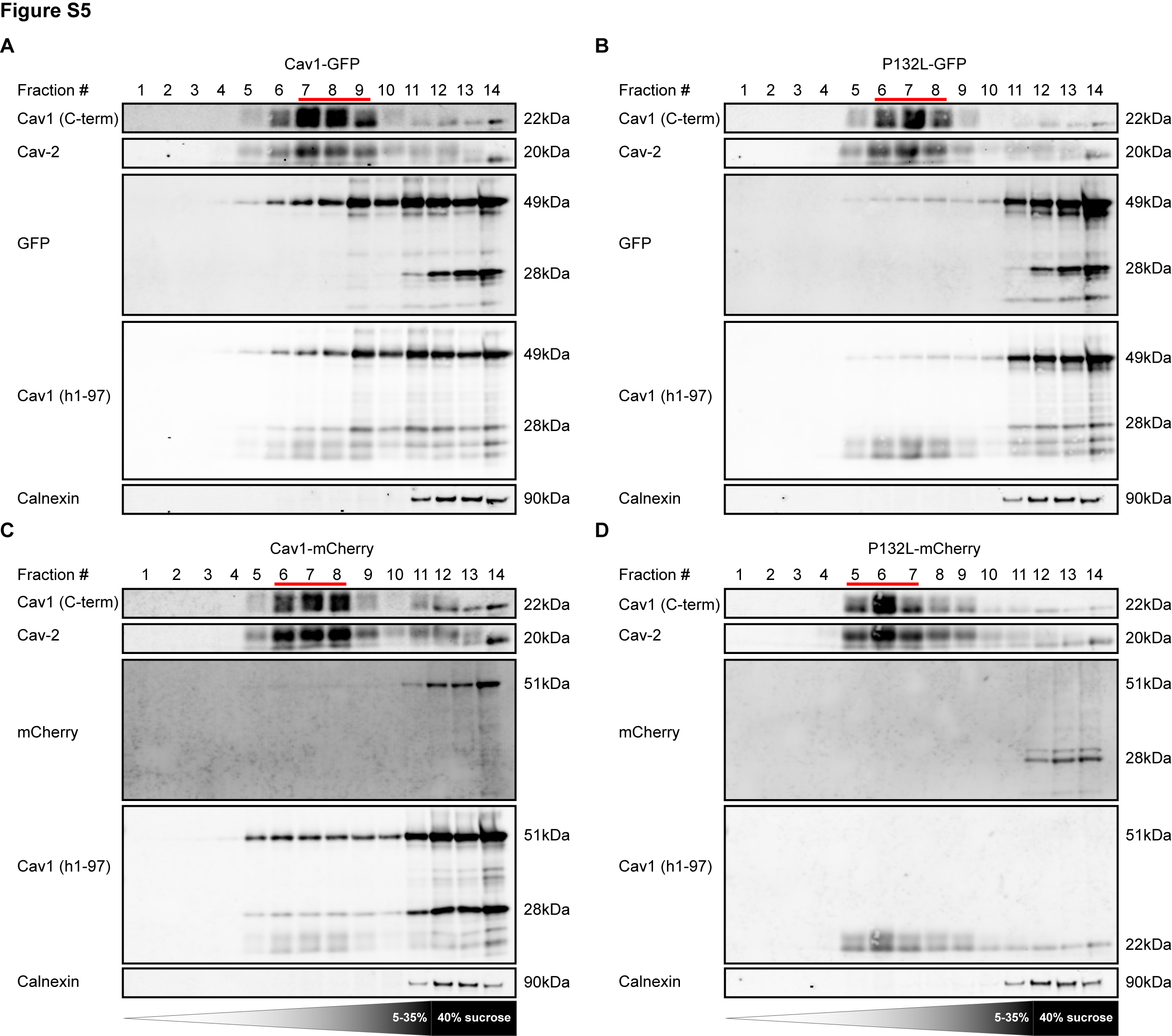
**

**Supplementary Figure 5 (associated with Figure 10). The affinity of overexpressed Cav1 for detergent resistant membranes differs as a function of the tag.** DRMs were isolated from COS-7 cells transiently expressing **(A)** Cav1-GFP, **(B)** P132L-GFP, **(C)** Cav1-mCherry, **(D)** P132L-mCherry and fractions were analyzed by SDS-PAGE/Western blotting. The position of endogenous caveolin-1 in DRMs is indicated for each blot with a red line.

This figure shows full blots for Figure 10, which include the degradation products for FP tagged Cav1 and P132L.
